# Comprehensive Analysis of Acetylation-Related lncRNAs and Identified AC099850.3 as Prognostic Biomarker in Non-Small Cell Lung Cancer

**DOI:** 10.1155/2021/4405697

**Published:** 2021-10-12

**Authors:** Junliang Zhou, Mingyan Zhang, Huanhuan Dong, Meiqi Wang, Yue Cheng, Shuqing Wang, Wenbo Ma, Hui Xu

**Affiliations:** Department of Clinical Laboratory, Harbin Medical University Cancer Hospital, 150 Haping Road, Harbin 150081, China

## Abstract

The present study aimed to analyze the effects of acetylation-related lncRNAs in non-small-cell lung cancer (NSCLC). A total of 399 differentially expressed lncRNAs (DElncRNAs) have been identified between 497 NSCLC tissues and 54 normal tissues in the TCGA database, and 105 of which were correlated with acetylation regulators. By using univariate cox regression analysis and combining it with clinical prognosis information, 12 prognostic-related lncRNAs were selected for the subsequent analysis. The NSCLC patients were divided into two subgroups (cluster 1 and cluster 2) by clustering software, and immunocyte infiltration analysis, microenvironmental analysis, and clinical relevance analysis were performed between the two subgroups. A risk model was also built to further assess the prognosis value of prognostic-related lncRNAs in NSCLC patients. We found that AC099850.3 was significantly higher in both cluster 1 and high-risk subgroups, which may serve as a potential biomarker for the prognosis of NSCLC patients. Then, based on ceRNA competition mechanisms, the pathway enrichment of 105 acetylation-related lncRNAs was conducted by GO and KEGG analyses. We found the acetylation-related lncRNAs were primarily enriched in MAPK and EGFR signaling pathways, which were closely associated with NSCLC development. Finally, we validated the expression levels of AC099850.3 in NSCLC tissues and adjacent non-cancerous tissues and confirmed that AC099850.3 was significantly highly expressed in NSCLC tissues and cells. These results may provide clues for our understanding of the role of acetylation-related lncRNAs and valuable information for future clinical diagnosis and prognosis in NSCLC patients.

## 1. Introduction

As a malignant tumor, lung cancer has seriously threatened human health. According to the survey in 2020, lung cancer was a leading cause of cancer-related mortality and the second common diagnosed cancer worldwide, with a mortality rate of 18.0% and the incidence rate of 11.4% [1]. Lung cancer has a low five-year survival rate and is often diagnosed at an advanced stage. Some clinical research showed that most early-stage NSCLC patients do not need to receive adjuvant chemotherapy after surgery, as the side effect was served and exceeding the benefit of survival. Therefore, exploring new molecular diagnostic and prognosis markers will be helpful for NSCLC patient therapy.

Acetylation is the process of adding acetyl (CH_3_CO–) to nitrogen, oxygen, and carbon atoms from organic compound molecules. The most common type is histone acetylation. Histone acetylation regulators include three major elements: histone acetyltransferases (writers), deacetylases (erasers), and specific enzymes (readers). Histone acetyltransferases (HATs) were composed of GANT family, MYST family, and ORPHAN family, which can regulate gene transcription by acetylating histone proteins or other non-histone substrates [2]. According to the presence of a conserved deacetylase domain and specific cofactors, HDACs are divided into the histone deacetylase family (HDAC1-11) and the sirtuin protein family (SIRT1-7) [3]. HDACs have a deacetylation effect that accelerates the degradation of the transcription factors [4]. HATs and HDACs mediate histone acetylation and deacetylation, respectively [5]. BRD4 is a specific enzyme that can be bound and identified.

Long non-coding RNA (lncRNA), a type of non-protein-coding RNA with a length longer than 200 nucleotides, has been reported to be involved in many life activities, such as genetic and epigenetic regulation, cell proliferation, and cell cycle regulation, which becomes a hot topic of genetic research. The research also showed that lncRNA played an important role in the occurrence and development of NSCLC. Particularly, acetylation-related lncRNAs play a vital role in cancer development, such as SNHG14 in HCC [6], MIR22HG in liver cancer [7], EIF3J-AS1 in colorectal cancer [8], and lncRNA-JADE in breast tumorigenesis [9]. However, due to the limitations of methods, techniques, and actual situation, some acetylation-related lncRNAs have not yet been found.

In our present study, we investigated the role of acetylation-related lncRNAs in NSCLC for the first time. We used the expression data of acetylation-related lncRNAs of LUAD in the TCGA database and identified 12 acetylation-related lncRNAs with prognostic values. Then, the LUAD patients were divided into two subgroups using the clustering software to analyze the clinical relevance and prognostic value of acetylation-related lncRNAs and further explore the relationship between acetylation-related lncRNAs prognostic signature and immune cell infiltration. We found that AC099850.3 may serve as a potential biomarker for the prognosis of NSCLC patients. Based on ceRNA competition mechanisms, we found that the acetylation-related lncRNAs were primarily enriched in MAPK and EGFR signaling pathways, which were closely associated with NSCLC development. The expression levels of AC099850.3 were significantly higher in NSCLC tissues and cells, which were consistent with the TCGA database. Our work may provide potential clues to increase the prognosis accuracy of NSCLC patients.

## 2. Materials and Methods

### 2.1. Data Acquisition

The RNA transcriptome and clinically relevant data for LUAD were downloaded from The Cancer Genome Atlas (TCGA) database (https://cancergenome.nih.gov/). According to the published literature in PubMed (https://pubmed.ncbi.nlm.nih.gov/), a total of 28 acetylation regulators were collected. The expression data for these 28 RNA transcriptomes were extracted using the R package. It paves the way for the next analysis of acetylation-related lncRNAs.

### 2.2. Bioinformatic Analysis

We extracted the expression data from the TCGA database and analyzed the differentially expressed lncRNAs (DElncRNAs) between 497 NSCLC tissues and 54 normal tissues in the data set. A total of 399 DElncRNAs have been identified by using the R package “limma” based on the standards of |log_2_ (Fold change)| > 1 and *P* < 0.05 and then conducted the Pearson correlation analysis to identify acetylation-related lncRNAs (|Pearson *R*| > 0.4 and *P* < 0.001). A total of 105 lncRNAs were identified to be correlated with acetylation regulators. Combined with clinical prognosis information, 12 prognostic-related lncRNAs were selected from 105 acetylation-related lncNAs in the TCGA data set using univariate Cox regression analysis. And then, the expression levels of 12 acetylation-related lncRNAs were analyzed in 497 tumor patients and 54 normal lung tissues using the Limma package, and then ggplot2 was used to visualize them. The middle line of the rectangle represents the median value Q2. The rectangular length scale represents the range from the bottom quarter to the upper quarter. The upper edge of the rectangle is the upper quartile Q3, and the lower edge is the lower quad Q1. Points that are outside the limit range are called abnormal values. The horizontal axis length of the graph represents the data distribution of an ordinate position, and the length of the vertical axis represents the degree of dispersion of the data.

Then, we removed 54 samples of normal tissue and grouped 497 cancer tissues using the “ConsensusClusterPlus” package to perform two clustering subgroups. The clinically relevant heat map was drawn using R packages “pheatmap” based on clustering groups. The package “corrplot” was used to plot the correlation of 12 prognostic-related lncRNAs. The “CIBERSORT” software package is designed to understand the proportion of immune cells in tumor tissue samples. We calculated the expression level of immune cells in each tumor sample using the “CIBERSORT” software package and then compared the differences in immune cells in the 2 cluster groups. At the same time, it paves the way for the subsequent microenvironmental analysis of tumors. A risk assessment model is constructed using 5 lncRNA of 12 prognostic-related lncRNAs, which are AC090559.1, AC099850.3, AC024075.2, AC005034.3, and HCG18. We used the FPKM value of 5 lncRNAs to score each tumor sample and divide it into 2 subgroups of high and low risk based on the median. Next, a total of 105 acetylation-related lncRNAs are used for the analysis of functional and pathway enrichment, including Gene Ontology (GO) Biological Processes and Kyoto Encyclopedia of Genes and Genomes Pathway (KEGG pathway) analysis. The “clusterProfiler,” “org.Hs.eg.db,” “enrichplot,” and “ggplot2” package were used in the GO and KEGG analysis to indirectly observe each key genes, functional module, and pathways of 105 acetylation-related lncRNAs.

### 2.3. Sample Collection

A total of 10 paired NSCLC tissues and adjacent non-cancerous tissues were collected from Harbin Medical University Cancer Hospital. All fresh tissues were stored immediately after root removal at −80°C. Our study was accepted by the ethics committee of Harbin Medical University Cancer Hospital.

### 2.4. Quantitative Real-Time PCR (qRT-PCR)

The expression levels of lncRNA AC099850.3 were measured by quantitative real-time PCR (qRT-PCR). The primers used for qRT-PCR were as follows: AC099850.3 forward, 5′-CTGGAGTGGCAGTGTTGCAATC-3′ and reverse, 5′-GGTGACGCACACCTGTA GTCC-3′, GAPDH 5′-GAAGGTGAAGGTCGGAGTC-3′, and 5′-GAAGATGGTGAT GGGATTTC-3′. cDNA was synthesized by PrimeScript™ RT Reagent kit (Takara) according to the manufacturer's instructions. cDNAs were quantified by qRT-PCR amplification using an SYBR® Green Premix Ex Taq II (Takara) on Applied Biosystems (Carlsbad, CA, USA) 7500 system with GAPDH as the internal control. Expression levels of each gene were analyzed by the 2^−ΔΔCt^ method, and each sample was tested in triplicate.

### 2.5. Statistical Analysis

Univariate Cox regression analysis was used to calculate the hazard ratio of 12 DElncRNAs. The log-rank test method and the Kaplan–Meier curve were used to compare the OS of clustered subgroups (clusters 1 and 2) and risk subgroups (high/low risk). In the TCGA data set, we also used *t*-tests to compare the expression levels of age, sex, phased, T-state, M-state, and N-state in each subgroup of the NSCLC sample. The OS of patients were assessed through area under the curve (AUC) values of various predictors (age, gender, WHO grade, and risk score) by using the R package “timeROC.” The independent prognostic value of acetylation-related lncRNAs for OS was assessed using univariate and multivariate Cox regression analysis. On the issue of data processing, we deleted the missing data in the downloaded data set. AC099850.3 expression levels of NSCLC tissues and cell lines were analyzed using the two-tailed paired *t*-test and were calculated using mean with SD. All data are statistically analyzed using the R 4.0 version and SPSS software, and *P* < 0.05 is statistically significant.

## 3. Results and Discussion

### 3.1. Results

#### 3.1.1. Identification of Acetylation-Related lncRNAs in NSCLC Patients

**Figure 1:**
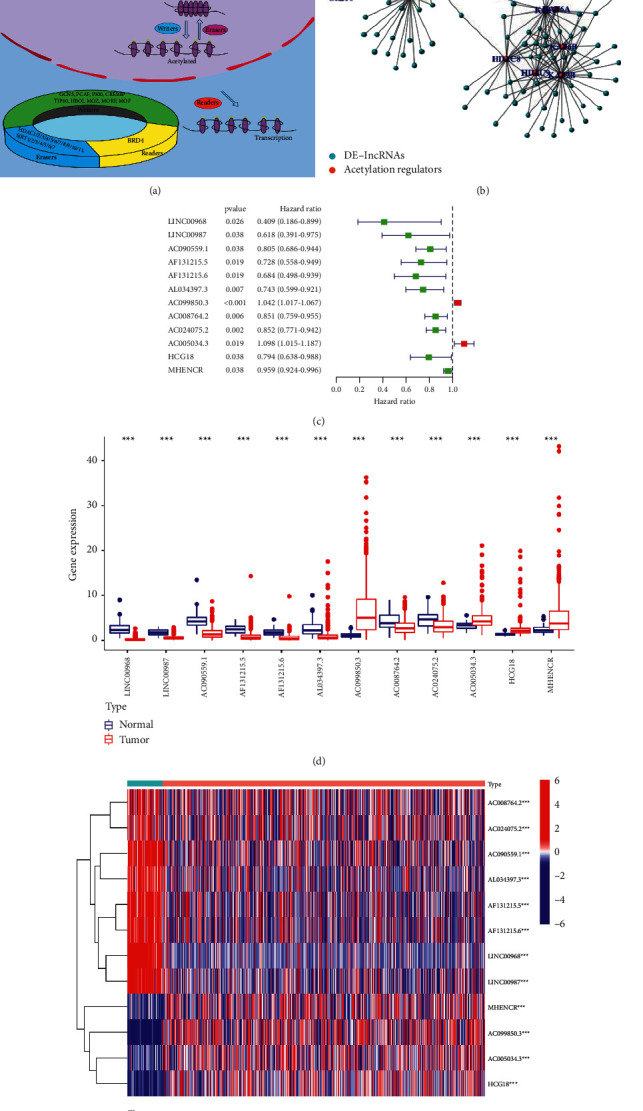
The landscape of acetylation-related lncRNAs in NSCLC patients. (a) The acetylation gene is divided into three main categories, including readers, writers, and erasers. (b) Network diagram of acetylation-related lncRNA. The acetylation regulators are “red,” and different lncRNAs are “blue” in lung cancer. (c) The process of building the signature containing 12 acetylation RNA. The hazard ratios (HR) and 95% confidence intervals (CI) were calculated by univariate Cox regression. (d and e) Expression of acetylation-related lncRNA in lung cancer and normal tissue.  ^*∗*^*P* < 0.05,  ^*∗∗*^*P* < 0.01, and  ^*∗∗∗*^*P* < 0.001.

First, we extracted the expression data from the TCGA database and analyzed the differentially expressed lncRNAs (DElncRNAs) between 497 NSCLC tissues and 54 normal tissues in the data set. We identified 399 DElncRNAs and then conducted the Pearson correlation analysis to identify acetylation-related lncRNAs. By analyzing the correlation between DElncRNAs and acetylation regulators, we obtained the acetylation regulators and acetylation-related lncRNA network (Figures 1(a) and 1(b)). We identified 105 acetylation-related lncRNAs that were correlated with the expression levels of acetylation regulators. Then the prognostic information was subject to univariate Cox regression analysis using the survival coxph function of the R package in the TCGA data set. Eventually, we identified 12 lncRNAs that were correlated with the overall survival (OS) of NSCLC patients, and we defined them as prognostic-related lncRNAs. Our results showed that NSCLC patients with low expression levels of LINC00968 (HR = 0.409; 95% CI = 0.19–0.90) and LINC00987 (HR = 0.618; 95% CI = 0.40–0.98) had better OS rates, and with high expression levels of AC005034.3 (HR = 1.098; 95%CI = 1.02–1.19) and AC099850.3 (HR = 1.042; 95%CI = 1.02–1.07) had a poorer lifetime (Figure 1(c)). The expression levels of 12 prognostic-related lncRNAs in the TCGA data set were shown in Figures 1(d) and 1(e). We found that most of the down-regulated lncRNAs in NSCLC patients were correlated with better OS rates, and vice versa, while two upregulated lncRNAs HCG18 and MHENCR in NSCLC patients were also correlated with better OS rates.

#### 3.1.2. Consensus Clustering of Acetylation-Related lncRNAs

**Figure 2:**
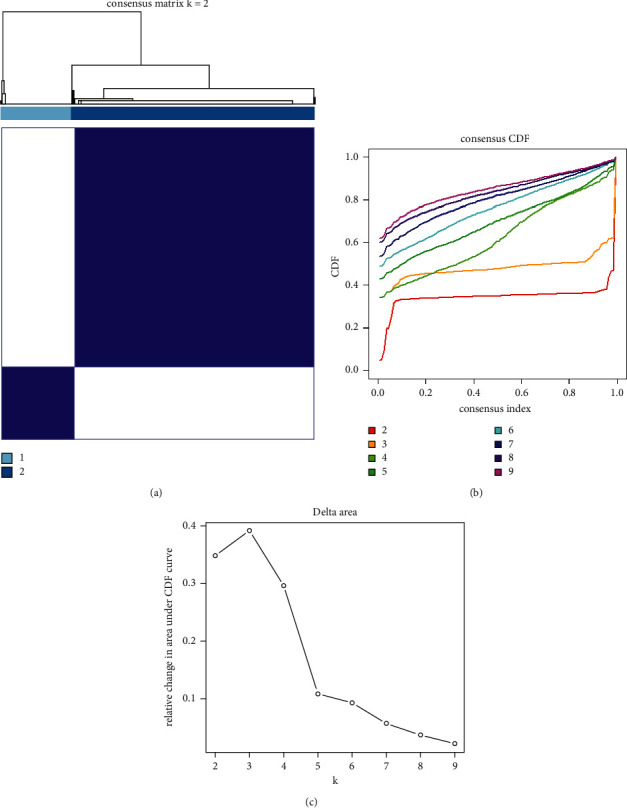
Consensus clustering of acetylation-related lncRNAs identified two clusters of NSCLC: (a) consensus clustering matrix for *k* = 2; (b) cumulative distribution function (CDF) for *k* = 2–9, clustering is stable when the CDF value is equal to 2; and (c) the diagram of relative change in area under CDF curve when *k* = 2–9. The comprehensive analysis found that it was better to divide into two subgroups.

Consensus clustering analysis was performed to group 497 NSCLC tissues using the Consensus Cluster Plus package. Based on the expression of acetylation-related lncRNAs, *K* = 2 had the smallest CDF value and the highest correlation within groups; therefore, the samples were divided into two groups for subsequent analysis (Figures 2(a)–2(c)).

#### 3.1.3. Differential Clinicopathological Features and Overall Survival of NSCLC Patients in Cluster 1 and 2 Subgroups

**Figure 3:**
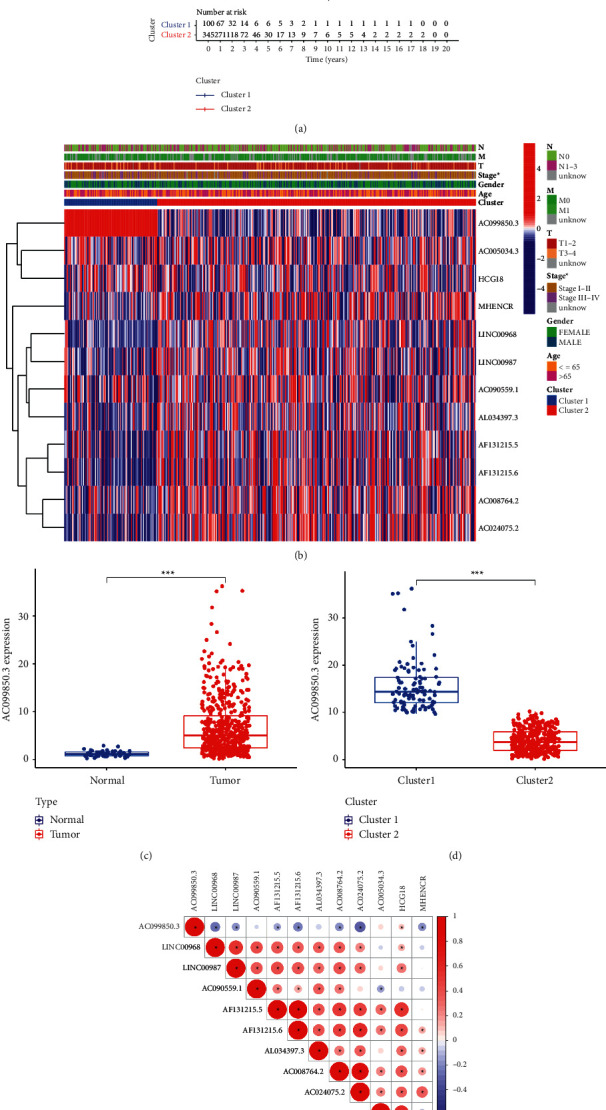
Differential clinicopathological features and overall survival of NSCLC patients in the two cluster subgroups. (a) Kaplan–Meier overall survival (OS) curves for 497 lung cancer patients from the TCGA database. The cluster 1 subgroup is noted with blue, and the cluster 2 subgroup is noted with red. (b) Heatmap and clinicopathologic features of the two cluster (cluster 1 and 2) subgroups and the expression of partial acetylation-related lncRNA in two subgroups. (c) The expression of lncRNA AC099850.3 in 54 normal tissues and 497 cancer samples. (d) The expression of lncRNA AC099850.3 in the two clusters (clusters 1 and 2). (e) Spearman correlation analysis of the correlation between 12 lncRNAs in lung cancer.  ^*∗*^*P* < 0.05 and  ^*∗∗*^*P* < 0.01.

We analyzed the clustering results and clinically relevant information. Kaplan–Meier curves were used to compare the OS of clusters 1 and 2. We found that the OS in the cluster 1 subgroup was significantly shorter than that in the cluster 2 subgroup (Figure 3(a)). In addition, lncRNA with a better prognosis was mainly concentrated in the cluster 2 subgroup, while lncRNA with a poor prognosis was mainly concentrated in the cluster 1 subgroup, especially lncRNA AC099850.3 in the cluster 1 subgroup (Figure 3(b)). Thus, lncRNA AC099850.3 is more likely related to the malignancy of NSCLC patients. Then, we visualized its expression and found lncRNA AC099850.3 was significantly highly expressed in both the tumor group and the cluster 1 subgroup (Figures 3(c) and 3(d)). The correlation between 12 prognostic-related lncRNAs was shown in Figure 3(e). And lncRNA AC099850.3 had a different degree of negative correlation with most other lncRNAs (Figure 3(e)).

#### 3.1.4. Immunocyte Infiltration Analysis and Tumor Microenvironment Score of NSCLC in Cluster 1 and 2 Subgroups

**Figure 4:**
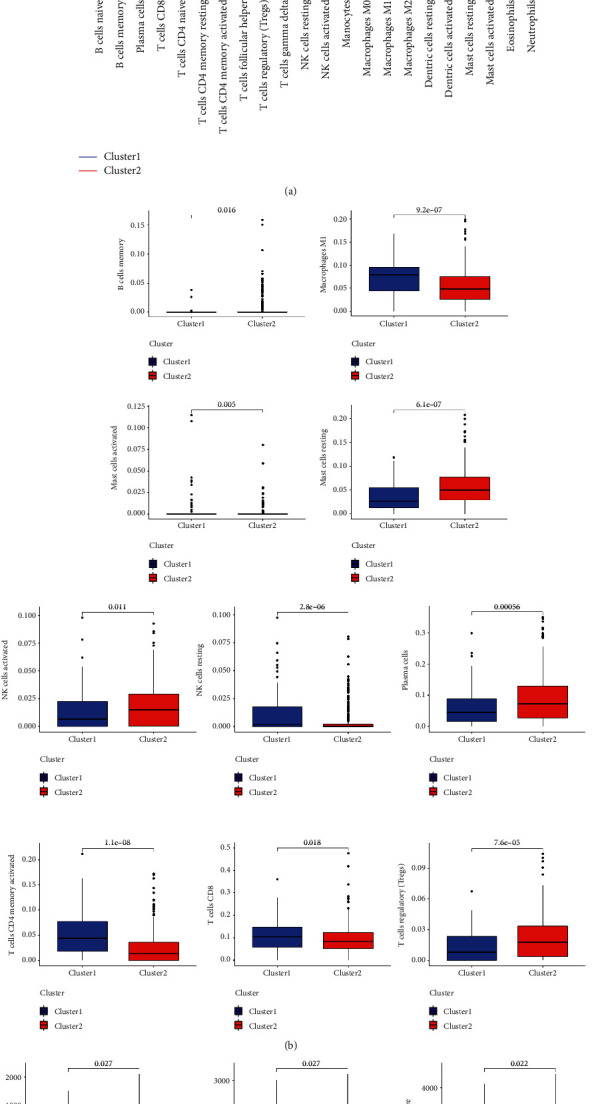
Differential immunocyte infiltration analysis and tumor microenvironment score of NSCLCs in the cluster 1 and 2 subgroups: (a) comparison of immune cell content in samples from two subgroups; (b) the expression of differences in immune cells; and (c–e) ImmuneScores, StromalScores, and ESTIMATEScores in the cluster 1 and 2 subgroup. *P* < 0.05 is statistically significant.

To better understand the clustering result, we performed immunocyte infiltration analysis and tumor microenvironment score estimation on samples from two subgroups. First, each tumor sample was analyzed using the CIBERSORT package to get a percentage of each immune cell's content in each tumor sample. Using the grouping of clusters, the immune cell content of the two subgroup samples was analyzed by the limma package, which was then visualized by the vioplot package (Figure 4(a)). At the same time, 10 immunocytes with significant differences were visualized in the form of box diagrams (Figure 4(b)). Our results showed that the cluster 1 subgroup had weak immunity and was more likely to lead to malignant progression of NSCLC. Then, the estimate package was applied to perform tumor microenvironmental analysis on each sample. Our estimate package evaluated each sample primarily through ImmuneScores, StromalScores, and ESTIMATEScores. We found that the Cluster 1 subgroup had a lower immune score, less stromal cell content, and higher tumor cell purity (Figures 4(c)–4(e)). Therefore, the cluster 1 subgroup was more harmful than the cluster 2 subgroup.

#### 3.1.5. Clinicopathological Features and Prognostic Model Construction in NSCLC

**Figure 5:**
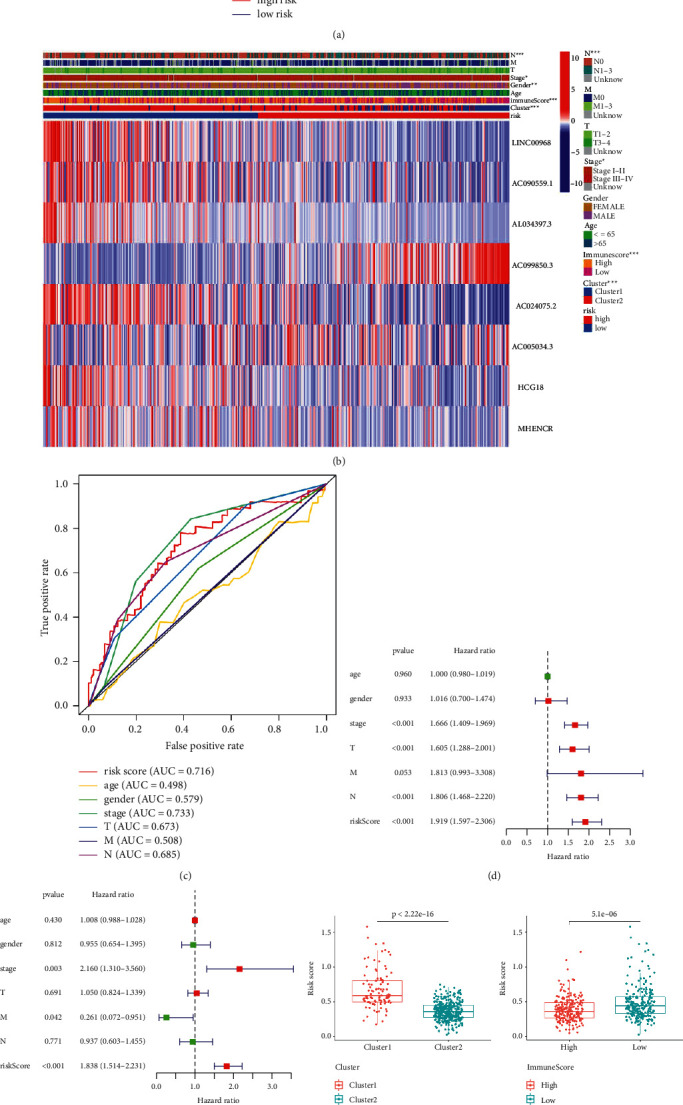
Risk scores are closely related to clinicopathological features and prognostic in NSCLC. (a) Kaplan–Meier overall survival (OS) curves for high- and low-risk patients. (b) The heatmap shows the expression levels of partially acetylation-related lncRNA in two risk groups (low and high) of NSCLC patients. And, the clinicopathological features were compared between the low- and high-risk groups. (c) The ROC curve shows the predictive efficiency of predictors, which includes various clinical characteristics and the risk score, for the survival of NSCLC patients. (d) Univariate Cox regression analysis shows that the relation between clinicopathological factors (including the risk score) and overall survival of patients in the TCGA data sets. (e) Multivariate Cox regression analysis. (f) Difference analysis of risk score in the clusters 1 and 2 and ImmuneScore group of low and high. (g) The expression analysis lncRNA AC099850.3 in high- and low-risk groups.  ^*∗*^*P* < 0.05,  ^*∗∗*^*P* < 0.01, and  ^*∗∗∗*^*P* < 0.001.

To better recognize the character of acetylation-related lncRNAs in NSCLC, we built a risk model and then divided NSCLC patients into two subgroups of low and high risk based on the medium risk score. The outcomes showed that patients had a poor five-year survival rate in the high-risk group (Figure 5(a)). Then, we systematically studied the expression of acetylation-related lncRNAs and the pathological characteristics of NSCLC patients in high- and low-risk subgroups (Figure 5(b)). Specially, the expression levels of lncRNA AC099850.3 were significantly higher in high-risk subgroup than in low-risk subgroup.

In addition, to better comprehend the relationship between risk scores and prognosis of patients with NSCLC, the ROC curve was a plot to predict OS rates in NSCLC patients. We found that risk score can predict its survival rate very well (AUC = 0.716; Figure 5(c)). Then, univariate and multivariate Cox regression analyses were conducted based on clinical pathology information of lung adenocarcinoma patients in the TCGA data set. The results showed that risk scores, gender, stage, T, M, and N status were all allied with OS (Figure 5(d)). When these predictors predicted it together, the risk score is still highly evaluative (Figure 5(e)). After that, we grouped the samples with the risk score into previous clusters (clusters 1 and 2) for a different analysis. The results showed that the risk score in the cluster 1 subgroup was higher than in the cluster 2 subgroup, which had a closer prognostic effect on patients with NSCLC. In addition, the risk score was lower in the high ImmuneScore subgroup and higher in the low ImmuneScore subgroup (Figure 5(f)). This echoes what we have received before.

Finally, we compared the different expression levels of lncRNA AC099850.3 in the high- and low-risk groups. The results showed that the expression of AC099850.3 was significantly higher in the high-risk subgroup than in the low-risk subgroup (Figure 5(g)).

#### 3.1.6. GO and KEGG Enrichment Analysis of Acetylation-Related lncRNAs

**Figure 6:**
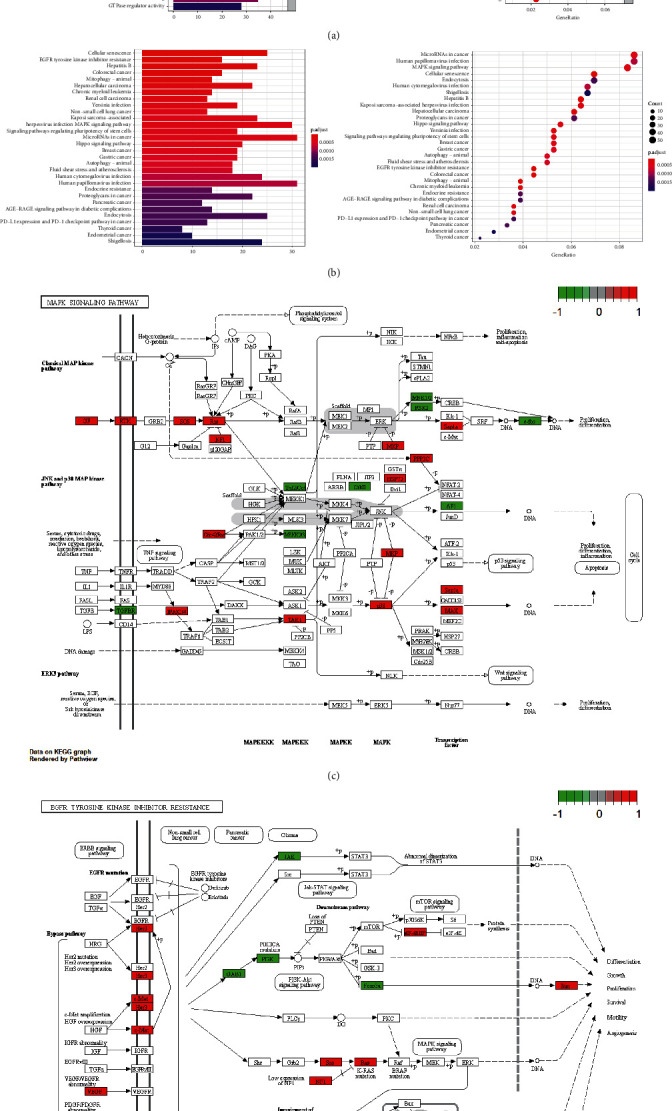
GO and KEGG enrichment analysis of acetylation-related lncRNAs: (a) GO enrichment analysis of acetylation-related lncRNAs; (b) KEGG enrichment analysis of acetylation-related lncRNAs; and (c and d) the network diagram of MAPK and EGFR signaling pathway. Red represents upward, and green represents downward.

To further explore the function of acetylation-related lncRNA in NSCLC and the signaling pathway that it may be involved in, the 105 differentially expressed acetylation-related lncRNAs were analyzed through GO and KEGG analysis. According to GO analysis (Figure 6(a)) results, acetylation-related lncRNAs were primarily enriched in positive regulation of the catabolic process, RAS protein signal transduction, and homeostasis of the number of cells in BP (biological process). In CC (cellular component), it is primarily involved in the composition of transcription regulator complex, cell-cell junction, and focal adhesion. And, it is primarily involved in molecular functions, such as protein serine/threonine kinase activity, transcription coregulator activity, and small GTPase binding in MF (molecular function). KEGG pathway enrichment analysis showed that the target genes of these acetylation-related lncRNAs were primarily enriched in the PI3K-Akt signaling pathway, MAPK signaling pathway, and microRNAs in NSCLC (Figure 6(b)). Meanwhile, based on the KEGG analysis, we obtained the network diagram of MAPK and the EGFR signaling pathway. It showed each signal node more clearly (Figures 6(c) and 6(d)).

#### 3.1.7. Validation of the Expression Levels of lncRNA AC099850.3 in NSCLC Samples

**Figure 7:**
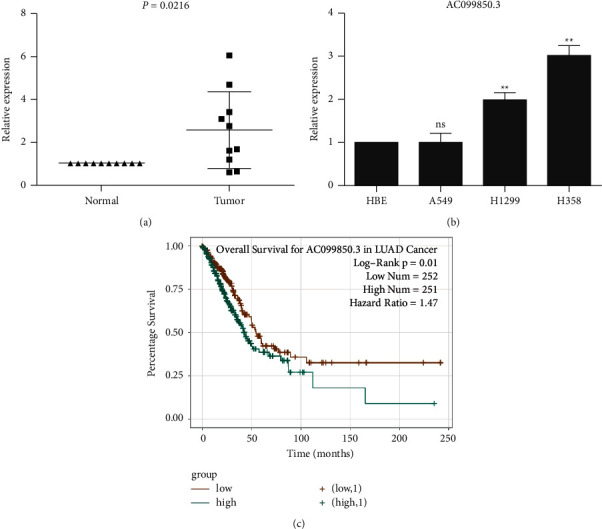
Validation of the expression levels of lncRNA AC099850.3: (a) RT-qPCR results showed the expression levels of the AC099850.3 in 10 pairs of non-small-cell lung cancer; (b) the expression levels of lncRNA AC099850.3 in lung cancer cell lines; and (c) the overall survival of AC099850.3 in LUAD cancer. *P* < 0.05 is statistically significant.

To validate the expression levels of lncRNA AC099850.3, qRT-PCR assays were performed in 10 paired NSCLC tissues and adjacent-noncancerous tissues. The expression levels of AC099850.3 were significantly higher in NSCLC tissues than in adjacent noncancerous tissues (*P* < 0.05; Figure 7(a)). In addition, we also tested the expression levels of the lncRNA in NSCLC cell lines and bronchial epithelial cell lines. The results showed that lncRNA AC099850.3 was highly expressed in NSCLC cell lines compared with normal bronchial epithelial cell line HBE (Figure 7(b)). Finally, to verify the results from the TCGA database, we used starBase v2.0 (http://starbase.sysu.edu.cn/panGeneSurvivalExp.php) for OS analysis. We found that the high expression levels of AC099850.3 had a lower OS rate compared with the low expression group (Figure 7(c)).

### 3.2. Discussion

Lung cancer is the leading cause of cancer-related death in the world. The five-year survival rate in lung cancer patients is approximately 15% in all phases [1]. The World Health Organization (WHO) classified lung cancer into two subtypes: non-small-cell lung cancer (NSCLC), which accounts for about 85% of all cases, and small-cell lung cancer (SCLC), which accounts for about 15% [10]. There are many ways to treat lung cancer, for example, conventional cisplatin-based chemotherapy for NSCLC gives moderate efficacy [11]; Afatinib can be used as a first-line treatment for patients with EGFR-positive mutations in NSCLC [12]; PD-L1 inhibitors joined with SBRT instead of conventional radiotherapy might fight against NSCLC, further achieving more favorable survival outcomes [13]. However, in the process of long-term treatment, primary and secondary drug resistance can occur in all patients who receive targeted therapy, leading to treatment failure [14, 15]. Although targeted therapy can prolong patients' survival time, considerable problems remain to exist in the process of clinical practice. Therefore, exploring new therapeutic targets and molecular mechanisms for lung cancer remains a challenging issue.

In recent years, a lot of attention has been given to the epigenetic modification of lncRNAs which play an important role in both cell differentiation and individual development in human diseases. Interestingly, much evidence has revealed the importance of the epigenetic modification of lncRNA in cancer formation and progression. Zhao et al. found that YTHDF1 plays a key role in regulating the cycle process and metabolism of HCC cells by analyzing liver cancer sample data in the TCGA database [16]. Zhao et al. found that LncRNA DLX6-AS1 sponsors malignant phenotype and lymph node metastasis in prostate cancer by inducing LARGE methylation [17]. Chen et al. showed that low expression of lncRNA H19 in EGFR-mutant lung cancer regulates the resistance of lung cancer to erlotinib [18]. Yan et al. indicated that lncRNA ADAMTS9-AS2 has a new function to promote temozolomide resistance by upregulating the FUS/MDM2 axis in GBM cells [19]. Zhang et al. also found that lncRNA SNHG14 promotes hepatocellular carcinoma progression via H3K27 acetylation-activated PABPC1 by PTEN signaling [6]. In this study, we explore the role of acetylation-related lncRNAs in NSCLC. Presently, studies have shown that epigenetic modification played a key role in the occurrence and progression of cancer. It is of great value to improve diagnosis or provide alternative treatment strategies by marking and screening genes related to epigenetic modification. The discovery that changes and destruction of the effects of epigenetic modification promoted drug resistance to primary or accessory therapy provided a new avenue for the finding of new therapeutic targets [20].

Acetylation modification, as a relatively rich internal modification in mammalian cells, has been verified to be associated with a variety of tumors. For example, Li et al. found that acetylation modification of HDAC inhibited GRP78 secretion in colon cancer cells [21]; Valdes-Mora et al. also found that acetylation of H2A.Z was related to the regulation of gene expression and its increase was associated with the activation of oncogenes [3]; Iseki H et al. found that parvoviral NS regulated host gene expression through histone acetylation, suggesting a possible mechanism of oncosuppression in thymic lymphoma [22].

In our present study, the DElncRNAs were analyzed between 497 NSCLC tissues and 54 normal tissues in the TCGA database, and 399 DElncRNAs were identified. By using Pearson correlation analysis, a total of 105 lncRNAs were identified to be correlated with acetylation regulators. Combined with clinical prognosis information, 12 prognostic-related lncRNAs were selected in the TCGA data set. Then we systematically analyzed the expression levels of 12 prognostic-related lncRNAs in 497 cases of NSCLC as well as their relationship to clinical pathological characteristics. Specially, lncRNA AC099850.3 was highly expressed in both the cluster 1 subgroup and the high-risk score subgroup, with the most significant difference among the four highly expressed lncRNAs in the tumor. Wang and Qin reported that lncRNA CTD-2510F5.4 (AC099850.3) was closely related to 15 hub genes and enriched in the progression of cell cycle regulation and cell replication. Silencing of CTD-2510F5.4 induced apoptosis in gastric cancer cells. Moreover, high expression of CTD-2510F5.4 exhibited significantly shorter median survival time (MST) of gastric cancer patients [23]. Through the above findings and our bioinformatic results, AC099850.3 was selected for further analysis in NSCLC patients. To the best of our knowledge, lncRNA AC099850.3 was reported as a candidate prognostic biomarker in NSCLC for the first time. Although the vast majority of functions are not yet known, we demonstrated its association with cancer pathology and malignant progression through extensive data analysis.

In addition, some results have been found in exploring the relationship between acetylation-related lncRNAs. For example, MHENCR, HCG18, and AC005034.3 were also highly expressed in lung cancer patients. It is controversial that better-surviving lncRNAs, MHENCR and HCC18, were highly expressed in NSCLC tissues compared with normal tissues. As we removed the normal tissues and retained the NSCLC tissues in the TCGA database, we speculate that these genes may aberrant high expressed in NSCLC tissues and protected the function of target genes that serve as tumor suppressor genes through some unknown mechanisms. Future studies will be conducted to explain this phenomenon. We then used cluster analysis to divide the tumor sample into groups by the expression of the acetylation-related lncRNAs. We found that only CDF values of two subgroups were best, although after dividing into three subgroups with a higher relative change in area under the CDF curve.

Interestingly, in analyzing the correlation among these acetylation-related lncRNAs, we found that there was a close correlation among them. For example, AC099850.3 showed a high negative correlation with LINC00968, AC024075.2, and MHENCR and showed a positive correlation with HCG18. LINC00968 can inhibit the progression of lung adenocarcinoma through the miR-21-5p/SMAD7 signal axis [24], and HCG18 acted as an oncogene in LUAD and enhanced LUAD progression by targeting the miR-34a-5p/HMMR axis [25]. Therefore, we speculate that whether there were some kinds of correlation mechanisms among them that should be further verified. Then, the CIBERSORT algorithm was used to calculate the abundance of various immune cells in each tumor patient in the clustered groups. Cluster 1's ImmuneScore was significantly lower than cluster 2, which indicated that the high expression of AC099850.3 at cluster 1 may be a potential influencer. Whether its high expression is associated with the abnormal expression of some immune cells is also worth exploring further. There are reports that genes highly expressed in tumors may have a better prognosis. For example, CXCL11 is primarily responsible for collecting various immune cells to local tissues and improved prognosis in colon cancer [26]. Therefore, genes highly expressed in tumors do not necessarily have a poor prognosis. We then built a risk model.

After building the risk model, a risk assessment was conducted of each tumor sample to obtain riskScore by applying futime, fustat, and the expression of acetylation-related lncRNAs in the risk model. We divided it into high- and low-risk groups based on riskScore. We found that riskScore (AUC = 0.716) was able to predict the time of survival better, with a higher hazard ratio (HR = 1.919). Currently, studies have shown that lncRNA promoted tumor development by participating in the process of acetylation. For example, stimulation of LncRNA TINCR by H3K27 acetylation endorses epithelial-mesenchymal transition (EMT) in breast cancer [27]. Acetylation-related lncRNAs are of great research significance in tumors. In KEGG analysis, genes associated with acetylation-related lncRNAs were primarily enriched in signaling pathways such as MAPK, EGFR, and so on. For example, IL-6R participates in the EMT process; key genes SOS1 and RAS are participants in the process of proliferation and differentiation in the KEGG diagram.

However, there were also some limitations in our study. We only use the TCGA database to analyze the acetylation-related lncRNAs, while the mechanisms of which were not elucidate. Further study should be conducted to illustrate the regulatory mechanisms of acetylation regulators and lncRNA AC099850.3 in the future. Recently, dysregulated, circulating lncRNAs can be detected in blood and urine, demonstrating the potential clinical value of lncRNAs as a diagnostic biomarker and prognosis indicator. Detecting the corresponding changes of AC099850.3 in peripheral blood of patients in NSCLC will be very interesting.

## 4. Conclusions

In conclusion, acetylation-related lncRNAs were differentially expressed in NSCLC and mainly enriched in MAPK and EGFR signaling pathways, some of which were correlated with the prognosis of NSCLC patients. We built a risk model and found that the risk score was of great prognostic value for predicting five years survival rate of NSCLC. We also found that lncRNA AC099850.3 was highly expressed in tumors and closely related to the development and procession of NLSCLC, which showed great prognostic value. This may provide some clues for further research on the mechanisms of the acetylation-related lncRNAs and a better understanding of the immune cell-related genes involved in LUAD development.

## Data Availability

The data used in this study were taken from The Cancer Genome Atlas (TCGA) database. It is an open access database of 33 types of cancer (https://cancergenome.nih.gov/).
